# Reference values for hand grip strength in the South Korean population

**DOI:** 10.1371/journal.pone.0195485

**Published:** 2018-04-06

**Authors:** Chung Reen Kim, Young-Jee Jeon, Moon Chan Kim, Taeheum Jeong, Woo Ram Koo

**Affiliations:** 1 Department of Physical Medicine and Rehabilitation, Ulsan University Hospital, University of Ulsan College of Medicine, Ulsan, South Korea; 2 Department of Family Medicine, Ulsan University Hospital, University of Ulsan College of Medicine, Ulsan, South Korea; Ehime University Graduate School of Medicine, JAPAN

## Abstract

**Objective:**

To establish reference values for hand grip strength (HGS) in a healthy Korean population and to identify the dependent anthropometric variables that affect HGS.

**Methods:**

Based on the sixth Korea National Health and Nutrition Examination Survey from 2014 to 2015, we analyzed the HGS data of 7,969 South Koreans. Individuals with specific chronic diseases and who reported poor subjective health status were excluded to ensure a healthy population sample. Means with standard deviations (SDs) and 95% confidence intervals were calculated for each 5-year interval starting from 10 years of age. To determine the relationship between HGS and anthropometric variables, we performed correlation analyses between HGS and height, weight, and body mass index (BMI). Additionally, based on these findings, the cut-off value for low HGS was presented by deriving -2SD values of healthy young adults as recommended by the European Working Group on Sarcopenia in Older People.

**Results:**

The mean age and BMI of men and women were 38.3 and 38.2 years and 23.8 and 22.4 kg/m^2^, respectively. Mean HGS of the dominant hand in men and women was 39.5 and 24.2 kg, respectively. The peak in HGS was at 35–39 years of age, after which HGS decreased in both sexes. HGS was significantly correlated with height, weight, and BMI. The cut-off values for weak HGS were < 28.9 and < 16.8 kg in men and women, respectively.

**Conclusion:**

These results provide useful reference values to assess HGS in patients who undergo hand surgery or who have various diseases that affect HGS. Moreover, a cut-off value for low HGS may help in defining sarcopenia among the Korean population.

## Introduction

Hand grip strength (HGS) is a measure of the maximum static force that a hand can squeeze using a dynamometer. It is widely used because it is easy and inexpensive to evaluate [1, 2]. This measure is used to diagnose hand function after hand injury, set treatment goals, and evaluate the outcome of hand surgery. The measurement of HGS is also useful as a predictor of health status [3, 4], muscular strength, nutritional status, and disability [5]. Longitudinal studies suggest that poor grip strength is predictive of increased mortality from cardiovascular disease and cancer in men, even when adjusted for muscle mass and body mass index (BMI) [6, 7]. There are also associations between HGS and multiple chronic diseases and multimorbidity in men and women after adjusting for confounding factors. There is a similar linear trend of association with the number of chronic diseases in men but not in women [8]. There is a positive correlation between HGS and normal bone mineral density in postmenopausal women [9].

There are variations in HGS depending on race, sex, and age [10, 11]; thus, it is necessary to determine standardized reference values. At present, HGS is measured for normal people in many countries and normative reference data dependent on sex and age are used in clinical practice [2, 12, 13]. In general, men have greater HGS than women at all ages [2] and HGS is higher in the right than the left hand in both sexes [12]. Furthermore, HGS increases from childhood through adolescence, peaks at age 35–40 years, and decreases thereafter. In developing countries, HGS is significantly lower than in developed countries [10]. Furthermore, HGS is known to be a reliable test, although the value obtained may differ among examiners, evaluation methods, and evaluation tools [14, 15].

Normative HGS data have been reported for South Korea; however, while the results are similar to those from other countries, the number of patients was small and there was insufficient consideration of other underlying diseases in previous studies. The Ministry of Health and Welfare annually evaluates and announces various health indicators for 10,000 people aged ≥ 10 years, and the latest Korean National Health and Nutrition Examination Surveys (KNHANES VI-2 and VI-3) conducted recently (2014–2015) included measurements of HGS. Based on the above, we aimed to identify the normative HGS data according to sex and age and examine the association of these data with some anthropometric indicators. Furthermore, we investigated the cut-off values of weak HGS, one of the defining components of sarcopenia in the older adult population of South Koreans.

## Materials and methods

### Data sources and study participants

We investigated data from the sixth Korea National Health and Nutrition Examination Survey (KNHANES), which was conducted by the Korea Centers for Disease Control and Prevention (KCDC) in 2014 and 2015. The KNHANES VI-2 and VI-3 studies measured HGS among participants aged ≥ 10 years, except for those who were excluded based on the following criteria: no hands, arms, or thumbs/ paralysis of hands/ cast on hands or fingers/ bandage on hands or wrist/ hand or wrist surgery in the prior 3 months/ pain, tingling, or stiffness in hands or wrist within the prior week.

Furthermore, people with any history of specific diseases such as osteoarthritis, rheumatic arthritis, chronic obstructive pulmonary diseases, liver cirrhosis, chronic kidney disease, diabetes, thyroid disease, coronary heart disease, cancer, or sequelae after stroke were excluded to ensure a healthy study sample. These specific diseases were shown to affect muscle strength in previous studies [16–18]. Additionally, participants who reported poor subjective health status were excluded in order to exclude possible coexisting diseases. Participants’ subjective health status was classified into three levels (poor, fair, or good) based on responses to the question, “How do you assess your own health status?”

A total of 7,969 participants were included from the initial 13,387 participants who were aged > 10 years after excluding 3,452 participants with coexisting diseases and 1,966 participants whose data were unavailable. Because the KNHANES was a weighted survey, 7,969 participants represented 62,740,203 total participants.

The KNHANES comprised three component surveys: a health interview, a health examination, and a nutrition survey. Trained medical staff and interviewers performed the health interviews and examinations. Information on health behaviors such as cigarette smoking was collected via self-reported forms. Information about medical conditions, education, income, perceived health status, and dominant hand was identified via face-to-face interviews. Health examinations consisted of anthropometric measures such as height, weight, and HGS. Each participant provided informed consent prior to inclusion in the study and KNHANES VI-2 and VI-3 was approved by the KCDC institutional review board (2013-12EXP-03-5C, 2015-01-02-6C).

### Measurement of HGS

Hand grip strength was measured with a digital grip strength dynamometer (TKK 5401 GRIP D; Takei, Japan), which measures between 5.0 and 100.0 kg of force and has an adjustable grip span. The minimum measurement unit is 0.1 kg. During the assessment, participants were asked to stand upright with their feet hip-width apart and to look forward with the elbow fully extended. The dynamometer was held by the testing hand in a neutral, comfortable position (not flexed or extended) with 90° flexion at the index finger. Participants performed three trials for each hand alternately, always starting with the dominant hand. Participants were instructed to squeeze the grip continuously with full force for at least 3 seconds. They were asked not to swing the grip dynamometer during the test and not to hold their breath. The time between each trial was approximately 60 seconds. The average of three trials for each hand was recorded for statistical analysis.

### Description of variables

Monthly household income and education were considered as the main indicators of socioeconomic status. Regarding educational attainment, the participants were asked for their highest level of educational qualification. This was classified into three educational categories: completion of elementary school, middle and high school, and post-secondary school. Household income was calculated based on equalized income (total household income divided by the square root of the number of household members) and classified into quartiles.

### Statistical analysis

The characteristics of the study participants were presented as means (standard error) or percentages (standard error). The mean and standard deviation (SD) of HGS and 95% confidence intervals (CIs) were calculated for each 5-year interval starting from 10 years of age. Correlation analysis was performed to determine the relationship between HGS and height, weight, or BMI. Additionally, the cut-off value for weak HGS was calculated by deriving the -2SD values of healthy young adults as recommended by the European Working Group on Sarcopenia in Older People (EWGSOP) [13, 17]. We defined “healthy young adults” by the 30–39 age group based on the findings of this study. Because the KNHANES was conducted using a complex sampling design and included weighted data following statistical guidance from the KCDC to ensure that the data collected represented the general Korean population, all analyses in this study were performed using sample weights. The sample weights were constructed for sample participants to represent the Korean population by accounting for the complex survey design [19]. Estimated proportions and their standard errors and mean values were calculated using the complex sampling procedure by adding the weighted values. All statistical analyses were conducted for men and women separately. Statistical analysis was performed using SPSS ver. 22.0 (SPSS Inc., Chicago, IL, USA). A two-tailed *p*-value < 0.05 was considered statistically significant.

## Results

The mean age of the participants was 38.3 and 38.2 years for men and women, respectively, and the mean BMI was 23.8 kg/m^2^ for men and 22.4 kg/m^2^ for women. Dominant HGS was 39.5 kg for men and 24.2 kg for women, and most men and women responded that their right hand was their dominant hand (89.4% for men, 90.0% for women) (Table 1).

**Table 1:** General characteristics of study participants.

	Male (*n* = 3,835, *N* = 33.4^a^)	Female (*n* = 4,134, *N* = 29.3^a^)
Age, years	38.3 (0.3)	38.2 (0.3)
Height, cm	170.8 (0.2)	158.4 (0.1)
Weight, kg	69.8 (0.2)	56.3 (0.2)
BMI, kg/cm^2^	23.8 (0.1)	22.4 (0.1)
Right hand grip strength, kg	39.5 (0.2)	24.1 (0.1)
Left hand grip strength, kg	37.9 (0.2)	22.8 (0.1)
Dominant hand grip strength, kg	39.5 (0.2)	24.2 (0.1)
Dominant hand, %		
Right hand	89.4 (0.6)	90.0 (0.6)
Left hand	5.4 (0.4)	4.6 (0.4)
Both hands	5.2 (0.4)	5.4 (0.4)
Smoking status, %		
Never smoke	38.7 (0.9)	90.6 (0.6)
Ex-smoker	27.4 (0.9)	5.3 (0.4)
Current smoker	33.9 (0.9)	4.1 (0.4)
House income quartile, %		
1 (lowest)	9.2 (0.6)	12.0 (0.8)
2	23.7 (1.0)	23.4 (1.1)
3	32.9 (1.2)	32.5 (1.1)
4 (highest)	34.2 (1.4)	32.1 (1.4)
Education level, %		
≤ 6 years	15.3 (0.6)	18.4 (0.7)
7–9 year	11.3 (0.6)	11.8 (0.6)
10–12 years	36.1 (1.0)	33.9 (1.0)
≥ 13 years	37.2 (1.1)	35.9 (1.1)

Values are presented in mean (standard errors) or percentages (standard errors).

^a^*n* unweighted sample size, *N* weighted sample size in millions. BMI = body mass index.

The mean, SD, and 95% CIs of HGS of the right hand, left hand, and dominant hand were calculated at 5-year intervals starting from 10 years of age. The mean HGS of the dominant hand in 10–14-year-old males was 23.2 (SD, = 8.8; 95% CI, = 22.3–24.1). For men > 80 years old, the mean dominant HGS was 26.2 (SD = 6.8, 95% CI = 24.7–27.7) (Table 2).

**Table 2:** Hand grip strength according to age in males.

Age (years)	Right Hand Grip Strength (kg)		Left Hand Grip Strength (kg)		Dominant Hand Grip Strength (kg)	
	Mean (SD)	95% CI	Mean (SD)	95% CI	Mean (SD)	95% CI
10–14	23.1 (8.7)	22.2–24.1	21.9 (8.0)	21.1–22.7	23.2 (8.8)	22.3–24.1
15–19	36.8 (6.8)	36.0–37.5	35.0 (6.7)	34.3–35.8	36.7 (7.6)	35.8–37.6
20–24	40.0 (7.5)	38.9–41.1	37.9 (7.4)	36.9–38.9	40.1 (7.6)	39.0–41.1
25–29	42.1 (7.2)	41.1–43.1	40.1 (7.2)	39.1–41.2	41.8 (7.6)	40.7–42.8
30–34	44.4 (7.6)	43.3–45.4	42.1 (7.3)	41.1–43.1	44.2 (8.2)	43.0–45.4
35–39	44.7 (7.1)	43.8–45.6	42.5 (6.9)	41.6–43.4	44.7 (7.3)	43.8–45.6
40–44	44.1 (6.9)	43.3–45.0	42.4 (6.8)	41.6–43.3	44.0 (7.6)	43.1–45.0
45–49	42.4 (6.0)	41.6–43.3	41.1 (5.8)	40.4–41.9	42.5 (6.1)	41.7–43.3
50–54	41.1 (6.3)	40.3–41.9	39.8 (6.0)	39.0–40.5	41.1 (6.2)	40.3–41.9
55–59	39.1 (6.6)	38.3–40.0	37.9 (6.2)	37.1–38.6	39.3 (6.5)	38.4–40.1
60–64	38.2 (6.3)	37.3–39.1	36.7 (6.3)	35.8–37.6	38.2 (6.4)	37.3–39.1
65–69	35.8 (5.7)	34.9–36.7	34.5 (5.6)	33.7–35.4	35.6 (6.3)	34.7–36.6
70–74	32.1 (6.1)	31.0–33.1	31.2 (5.6)	30.1–32.2	32.1 (6.7)	30.9–33.3
75–79	29.4 (6.8)	28.0–30.7	28.9 (6.3)	27.6–30.1	29.4 (6.8)	28.1–30.7
≥ 80	26.2 (6.8)	24.6–27.7	25.5 (6.1)	24.1–26.8	26.2 (6.8)	24.7–27.7

SD = standard deviation, CI = confidence interval.

In females, the mean dominant HGS of 10–14-year-olds was 18.8 (SD = 5.0, 95% CI = 18.3–19.4). The HGS value was the highest at 35–39 years of age and then decreased thereafter. For women > 80 years old, the mean HGS of the dominant hand was 14.6 (SD = 4.2, 95% CI = 13.7–15.4) (Table 3).

**Table 3:** Hand grip strength according to age in females.

Age (years)	Right Hand Grip Strength (kg)		Left Hand Grip Strength (kg)		Dominant Hand Grip Strength (kg)	
	Mean (SD)	95% CI	Mean (SD)	95% CI	Mean (SD)	95% CI
10–14	18.8 (5.1)	18.2–19.4	17.7 (4.6)	17.1–18.3	18.8 (5.0)	18.3–19.4
15–19	24.1 (4.5)	23.5–24.7	22.7 (4.4)	22.1–23.3	24.2 (4.5)	23.6–24.8
20–24	24.2 (4.6)	23.5–24.8	22.8 (4.6)	22.2–23.4	24.2 (4.6)	23.6–24.8
25–29	24.3 (4.4)	23.7–24.9	22.8 (4.4)	22.2–23.5	24.4 (4.4)	23.8–25.0
30–34	25.8 (4.6)	25.2–26.3	24.2 (4.4)	23.7–24.8	25.8 (4.6)	25.3–26.4
35–39	26.5 (4.5)	26.0–27.0	25.1 (4.3)	24.6–25.6	26.5 (4.5)	26.0–27.0
40–44	25.8 (4.5)	25.3–26.3	24.6 (4.2)	24.1–25.1	25.8 (4.7)	25.3–26.3
45–49	25.7 (4.4)	25.2–26.2	24.0 (4.3)	23.5–24.5	25.6 (4.6)	25.1–26.2
50–54	25.5 (4.2)	25.0–26.1	24.2 (4.1)	23.7–24.7	25.6 (4.3)	25.0–26.1
55–59	24.2 (4.2)	23.6–24.7	22.6 (4.0)	22.1–23.1	24.2 (4.2)	23.6–24.7
60–64	23.5 (4.4)	22.8–24.2	21.8 (3.7)	21.3–22.4	23.6 (4.3)	22.9–24.2
65–69	22.3 (4.7)	21.4–23.1	21.1 (4.1)	20.4–21.8	22.3 (4.9)	21.5–23.1
70–74	20.7 (4.7)	19.7–21.6	19.6 (4.4)	18.7–20.5	20.5 (4.8)	19.5–21.5
75–79	17.7 (5.0)	16.5–18.9	17.0 (4.8)	15.8–18.2	17.6 (5.3)	16.3–18.9
≥ 80	14.6 (4.2)	13.8–15.5	13.9 (4.0)	13.1–14.7	14.6 (4.2)	13.7–15.4

SD, standard deviation, CI, confidence interval.

**Fig 1:**
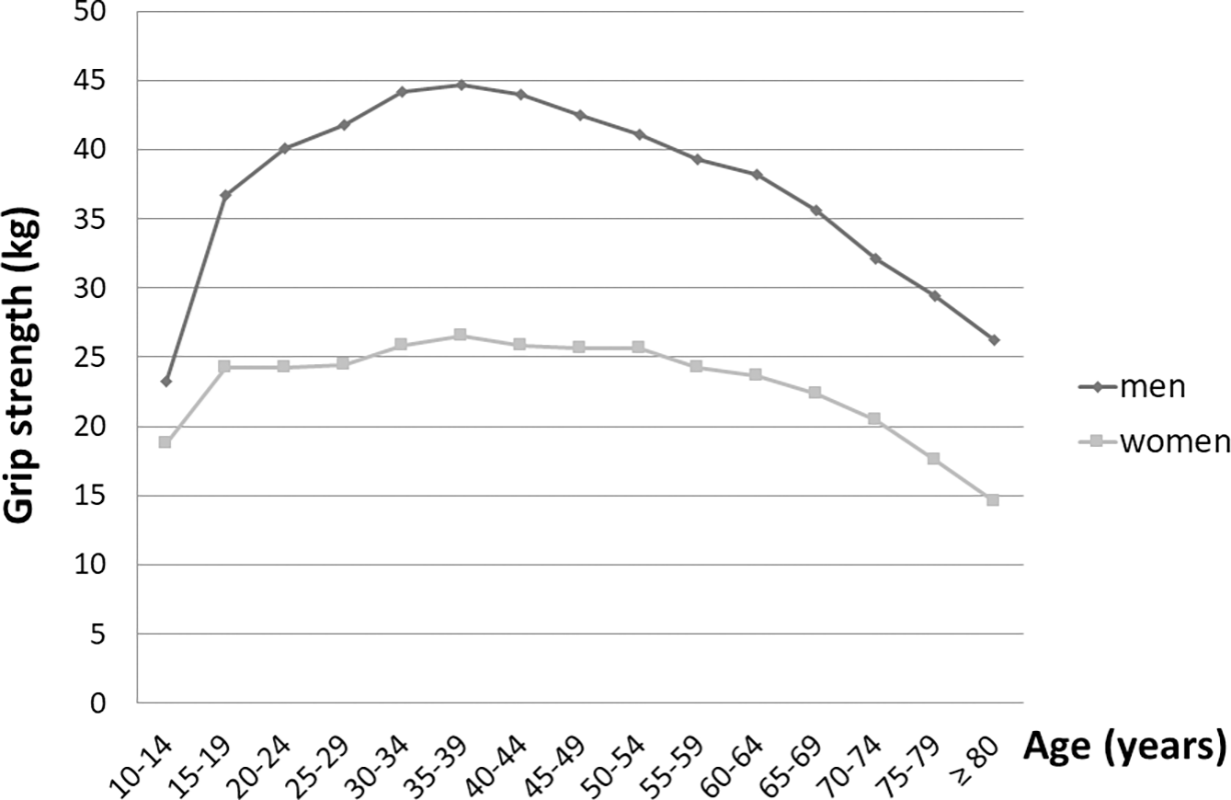
Mean hand grip strength of the dominant hand in males and females.

HGS in men and women reached its peak at 35–39 years of age and then decreased. There was broad maintenance around the peak, which was at 30–44 years in men and 30–54 years in women (Tables 2 and 3, and Fig 1).

There was a significant correlation between HGS and height, weight, and BMI regardless of sex, and the correlation coefficient between HGS and height was the highest among the three indices. In the analysis of age divided into < 20, 20–64, and ≥ 65 years, the correlation coefficient between HGS and height was the highest (0.753) for males aged 10–19 years. The correlation coefficients between HGS and height were the highest, except for men and women aged 20–64 years and men aged ≥ 65 years. In men aged > 65 years, the correlation coefficients were 0.312 between HGS and height and 0.345 between HGS and weight (Table 4).

**Table 4:** Correlation of hand grip strength with height, weight, and body mass index according to age.

Variable	Sex	Age group (years)			
		Total	10–19	20–64	≥ 65
Height	Male	0.562^a^	0.753^a^	0.312^a^	0.312^a^
	Female	0.446^a^	0.605^a^	0.271^a^	0.494^a^
Weight	Male	0.542^a^	0.661^a^	0.342^a^	0.345^a^
	Female	0.408^a^	0.601^a^	0.296^a^	0.358^a^
BMI	Male	0.362^a^	0.385^a^	0.222^a^	0.222^a^
	Female	0.213^a^	0.457^a^	0.169^a^	0.120^a^

BMI, body mass index.

^a^*p* < 0.01. The *p-*values were calculated using Pearson's correlation analysis.

The peak in HGS occurred in early life and was then followed by a period of broad maintenance prior to a decrease with aging (Fig 1). The 30–39 years group with an overlap between men and women showed a period of broad maintenance.

We evaluated young healthy adults in the 30–39 age group to calculate the cut-off value for weak HGS to define sarcopenia. The cut-off value for weak HGS was calculated by deriving -2SD values of healthy young adults in this age group. The cut-off values for weak HGS in sarcopenia were 28.9 kg and 16.8 kg for men and women, respectively (Table 5).

**Table 5:** Characteristics of the healthy young reference group (30–39 years).

	Male (*n* = 940, *N* = 9.7^a^)	Female (*n* = 1,190, *N* = 8.7^a^)
Age, years	36.9 ± 4.4	37.1 ± 4.3
Height, cm	174.4 ± 5.9	160.44 ± 5.3
Weight, kg	75.7 ± 12.2	58.0 ± 8.9
BMI, kg/cm^2^	24.8 ± 3.4	22.5 ± 3.3
Right hand grip strength, kg	44.4 ± 7.2	26.0 ± 4.5
Left hand grip strength, kg	42.4 ± 7.0	24.6 ± 4.3
Dominant hand grip strength, kg	44.3 ± 7.7	26.0 ± 4.6
Cut-off values for weak HGS in sarcopenia (less than 2SD), kg	28.9	16.8

The young reference group was healthy men and women aged 30–44 years.

Values are presented in mean ± standard deviation (SD).

^a^ *n* unweighted sample size, *N* weighted sample size in millions.

BMI = body mass index, HGS = hand grip strength, SD = standard deviation.

## Discussion

The purpose of this study was to establish HGS reference values according to age based on data from the 2014–2015 KNHANES. The reference values generated in this study are more reliable because this study was conducted on a larger scale than previous South Korean studies. Male and right-hand HGS values were higher than female and left-hand HGS values, respectively. The highest HGS value was observed in the 35–39 years age group in both men and women. HGS was strongly correlated with height, weight, and BMI. Among the three anthropometric factors, height showed the strongest correlation with HGS. In addition, cut-off values for weak HGS in sarcopenia were presented as -2SD values relative to a normal healthy population (the 30–39 age group), which revealed the peak HGS value in this study as recommended by the EWGSOP. The cut-off values for weak HGS in sarcopenia were 28.9 kg for men and 16.8 kg for women.

The findings that men had stronger HGS than women and that right-hand HGS was stronger in both sexes than left-hand HGS were consistent with previous studies [2, 12]. In this study, both men and women in the 35–39 years age range showed the highest HGS values. After this age, HGS gradually decreased. The peak age groups differed among this study and previous studies. However, the trends were similar. In previous studies, HGS was the strongest in men of the 20–30 age group and started to decrease in those in their 40s. In women, HGS was similar in the 20–40 age group and showed an accelerated decline from the age of 50 years [2, 11, 20–22]. The difference in HGS between the sexes was the lowest at 10–14 years of age. From the age of 15 years, HGS in men began to increase more rapidly than in women. At the peak HGS period, there was broad maintenance (age group 30–39 years), following which the HGS of men decreased quickly. In woman, HGS had a longer maintenance (age group 30–54 years) period; however, the HGS of women also weakened steeply after reaching the peak HGS value. This finding was similar to those of a previous study [13]. There were three key phases in HGS dynamics: an increase to the peak value in early adult life, broad maintenance through to midlife, and a decrease from the midlife value thereafter.

Starting at peak HGS, HGS in men declined at a rate of about 0.7–1.1% per age group between ages 40–64 years and declined at a rate of about 2.6–3.2% per age group from 65 years onwards. In woman, HGS declined gradually at a rate of about 0–0.7% per age group between ages 40~54 and declined dramatically at a rate of about 0.5–3.0% per age group thereafter. Age-related decreases in strength have been well documented in previous studies [13, 23]. The rate of decline in muscle strength may accelerate from the 6^th^ decade of life in men and women. However, the sex differences in HGS dynamics with aging are not well understood. In women, HGS declined dramatically in the 55–59 years group in the present study. Sex hormones are very important in explaining this sex-related difference [24, 25]. It has been demonstrated that muscle strength is similar in men and premenopausal women, whereas there is a steep decline in muscle strength at around the time of menopause onwards [26].

To determine the relationship between HGS and anthropometric variables, we performed correlation analyses between HGS and height, weight, and BMI. Earlier studies on the correlation between HGS and anthropometric data, such as BMI, height, and weight, yielded positive correlations [11, 27, 28]. In this study, height, weight, and BMI had the strongest correlations with HGS in both sexes. In addition, the correlation coefficient was higher in men than in women. Regardless of sex, the correlation between anthropometric indices and HGS was the highest in the 10–19 years age group. In addition, BMI was weakly correlated with HGS in men and women aged > 20 years. A recent study conducted in South Korea showed similar results; HGS was more strongly correlated with height and weight than BMI in both men and women [29]. Relative HGS was a more useful predictor of future cardiovascular health, disability, and mortality than absolute HGS in previous studies [30, 31]. In most previous studies, relative HGS was defined as the absolute HGS divided by BMI [30, 31] or weight [32, 33] but not by height. However, skeletal muscle mass adjusted by height correlates more strongly with muscle mass by taking into consideration body size than that adjusted by body weight or BMI [34, 35]. If relative HGS is used as an indicator of muscle function, considering body size, relative HGS adjusted for height among other anthropometric indices may be considered. However, further studies are needed to determine the usefulness of this index for predicting morbidity and mortality.

Differences in HGS, which reflects muscle strength as part of the defining criteria of sarcopenia, are observed according to race and ethnicity. Therefore, the cut-off values of South Koreans have not been established. The Asian Working Group of Sarcopenia (AWGS) suggested that the diagnostic criteria for sarcopenia involve muscle strength, skeletal muscle mass, physical performance, frailty status, and activities of daily living [16]. Weak HGS was defined as < 26 kg for men and < 18 kg for women or the lower 20th percentile for HGS of the study population by the AWGS [16]. The cut-off values for weak HGS in Japan were < 30.3 kg for men and < 19.3 kg for women, which represented < 25% of participants [36], and < 28.8 kg for men and < 18.2 kg for women, which represented < 20% of participants [37]. The cut-off values for weak HGS in China were < 28 kg for men and < 18 kg for women, which represented less than 20% of participants [38]. These cut-off values were similar to the cut-off values of men in the current study but higher than those in women. As in this study, a study of a Taiwanese population provided a cut-off value as recommended by the EWGSOP. The cut-off values for weak HGS were < 22.4 kg for men and < 14.3 kg for women. These cut-off values were about 25.4% lower in men and 27.4% lower in women than those of Caucasians [39] and were also lower than those calculated in the current study. These cut-off values are important in diagnosing sarcopenia and recognizing the need for treatment. As mentioned in the AWGS guidelines, a more accurate outcome-based study should be used. However, the -2SD values of healthy young adults may be useful indicators of sarcopenia until such cut-off values are obtained from outcome-based studies.

South Korea is one of the fastest aging countries and Asia is the fastest aging region in the world [40]. Older adults often face changes in body composition, which lead to a shift toward decreased muscle mass and increased fat mass, even in relatively weight stable, healthy individuals [41]. Sarcopenia, an age-related loss of skeletal muscle mass and function, is associated with the risk of various adverse health outcomes, including poor quality of life [36], frailty [42, 43], disability [44], and mortality [45]. Therefore, sarcopenia is an important clinical public health concern for older adults and the prevalence of sarcopenia is rising [46]. However, early diagnosis of sarcopenia risk can prevent some adverse outcomes. Physical activities, especially resistance training or compound exercise programs, seem to be good strategies for sarcopenia prevention [47, 48]. Hence, the cut-off values obtained in this study will be useful for screening and investigating the prevalence of the risk of sarcopenia in older adults.

A recent study [49] in South Korea reported HGS reference values. Yoo *et al*. recently reported normative values of HGS according to sex and age and cut-off values for weak HGS based on the 2015 KNHANES VI-3 data. However, we performed our analysis of these data to be representative of South Koreans using appropriate weighting steps and by including more participants than the previous study. Furthermore, to ensure that a healthy normal population was included in the current study sample, we excluded not only participants with diseases associated with muscle mass and muscle strength reduction but also those who reported poor subjective health status. This was because subjective health status is also known to be associated with morbidity and mortality [50, 51]. Therefore, the reference values and cut-off values obtained in this study may be more representative of the normal, healthy South Korean population and more useful in future studies related to chronic disease, hand surgery, and sarcopenia.

The present study has some limitations that should be discussed. First, the findings of this study cannot explain individual differences according to life course because we analyzed cross-sectional data. The difference in physical status between the healthy young and elderly groups increased dramatically in the last 20–30 years in South Korea due to rapid economic development and industrialization [52]. For this reason, cross-sectional age profiles of HGS may overestimate individual declines. Second, among the anthropometric indices, height had the strongest correlation with HGS. Therefore, we suggest that height should be considered as an adjustable parameter of relative HGS. However, further study is needed to determine if relative HGS adjusted for height explains the relationship between HGS and morbidity or mortality better than absolute HGS or relative HGS adjusted for other anthropometric indices. Finally, it was difficult to assess HGS comprehensively because various data, such as arm length, pinch grip power, and muscle mass, were not investigated. However, this study was meaningful because analyzed the differences in HGS according to age and sex using large-scale data representing the South Korean population and suggested cut-off values for weak HGS, which is one aspect of sarcopenia.

In conclusion, this study revealed the normal levels of HGS in a large-scale database representing the South Korean population. The correlations between HGS and anthropometric indices used for correcting for body size to determine relative HGS were analyzed. The correlation with height was the strongest. Therefore, we recommended relative HGS adjusted by height rather than BMI. Finally, we investigated cut-off values for low HGS in South Koreans. The cut-off values of the healthy young (mean−2.5SD) were < 28.9 kg for men and < 16.8 kg for women. These results could provide useful reference values to assess HGS in patients who undergo hand surgery or who have various diseases that affect HGS. Moreover, the cut-off value for weak HGS should be helpful in defining sarcopenia among the Korean population.
